# Toxic effects of VCD on kidneys and liver tissues: a histopathological and biochemical study

**DOI:** 10.1186/s13104-019-4490-y

**Published:** 2019-07-22

**Authors:** Shahin Ahmadian, Sepideh Sheshpari, Mahdi Mahdipour, Mohammad Pazhang, Pei-Shiue Jason Tsai, Mohammad Nouri, Reza Rahbarghazi, Mahnaz Shahnazi

**Affiliations:** 1 Women’s Reproductive Health Research Center, Tabriz University of Medical Sciences, Tabriz, Iran; 2 Department of Biology, Faculty of Science, Azarbaijan Shahid Madani University, Tabriz, Iran; 3 Department of Midwifery, Faculty of Nursing and Midwifery, Tabriz University of Medical Sciences, Shariati St., Tabriz, Iran; 4 Stem Cell Research Center, Tabriz University of Medical Sciences, Daneshgah St., Tabriz, Iran; 5 Department of Reproductive Biology, Faculty of Advanced Medical Sciences, Tabriz University of Medical Sciences, Daneshgah St., Tabriz, Iran; 6 Center for Developmental Biology and Regenerative Medicine Research, National Taiwan University/NTU, Taipei, Taiwan; 7 Department of Veterinary Medicine, School of Veterinary Medicine, National Taiwan University/NTU, Taipei, Taiwan; 8 Department of Applied Cell Sciences, Faculty of Advanced Medical Sciences, Tabriz University of Medical Sciences, Tabriz, Iran

**Keywords:** Kidneys, Liver, Premature ovarian failure, Side effects, Toxicity, Vinyl cyclohexene dioxide

## Abstract

**Objective:**

We explored detrimental effects of VCD on non-ovarian tissues such as kidneys and liver 14 days post-drug administration. Twelve rats were randomly assigned into two groups. In VCD group, rats received 160 mg/kgbw VCD intraperitoneally for 15 consequent days. Control rats were injected with VCD-free normal saline. At the respective time point, rats were euthanized, blood and tissue samples were collected. H&E staining was performed to evaluate pathological changes. Serum level of ALT, AST, creatinine and urea were also measured.

**Results:**

Histological analysis revealed hyperemia and follicular atresia in the ovaries, indicating successful POF induction in rats. In renal tissue, extensive tubular necrosis, focal hemorrhage, hyaline casts, and interstitial nephritis were observed. Analysis of hepatic tissue showed numerous hemorrhagic foci, chronic cholangitis, and hepatocyte necrosis, indicating apparent VCD toxicity of both hepatic and renal tissues. The biochemical evaluation revealed a tendency of increase in ALT, AST, creatinine, and Urea in VCD-treated rats; however, the values did not reach significant level. In conclusion, the induction of POF in rats by VCD correlates with renal and hepatic damages. Commensurate with data from this study, any conclusions from experiments based on VCD-induced premature ovarian failure rats should be reported with caution.

## Introduction

Infertility is one of the most important clinical problems globally reported in males and females [1, 2]. Various causes including structural, hormonal and environmental issues have been identified in relation to the fertility status [3]. Besides these issues, infertility could resulted from the treatments of some anti-cancer agents and the exposure to toxic substances [4–6]. In females, these conditions could lead to POF coincided with the depletion of ovarian follicles [7–9]. Several medical approaches are being undertaken for treating infertility such as hormone replacement therapy, gonadotropin releasing hormone agonist, and assisted reproductive technology. However, current therapeutic approaches are not satisfactory [10, 11].

VCD is one of the efficient chemical agents used commonly for the establishment of POF in animal models [12, 13]. VCD in principal is a byproduct of vinylcyclohexene commercially used as an adjuvant in epoxy resins and diepoxides [14]. Studies have shown that VCD has gonadotoxic properties and selectively target primordial and primary follicles [15]. It has been described that VCD accelerates the natural process of follicular atresia and cell apoptosis [12, 13]. KIT receptor and KITLG axis are critical in immature follicles and oocytes survival and VCD has been reported to modulate the KIT signaling pathway on the oocytes membrane causing atresia [15]; moreover, the attachment of granulosa cells-derived KITLG produced by membrane-bound tyrosine kinase receptors of oocytes activates protein kinase. In response to these modulations, KIT auto-phosphorylation is initiated via PI3K [15]. The promotion of PI3K could trigger PDK-1 phosphorylation and activates AKT protein kinase.

To induce POF, various doses of chemicals have been prescribed with different clinical outcomes. During the process of POF modeling, researchers have mainly focused on the effects of this chemical on ovaries; however, there are few reports regarding VCD effects on other organs especially, liver and kidneys. The objective of this study was to investigate the VCD side effects using POF animal model with special regard to evaluate the histopathological changes in liver and kidneys. We also monitored serum levels of ALT, AST, creatinine and urea following VCD administration. The results illustrated the significant pathological and histological changes in both organs indicated detrimental effects of VCD on hepatic and renal tissues. In spite of ALT and AST elevation in VCD-treated rats, no significant differences were found when compared to the control group. Presented results show the necessity for additional experimentations to find possible alternative substances for animal modeling of POF.

## Main text

### Materials and methods

#### Animal and ethical issues

The current experiment was performed in accordance with guidelines of the local ethics committee of Tabriz University of Medical Sciences. All phases of this study were coincided with “The Care and Use of Laboratory Animals (NIH Publication No. 85-23, revised 1996)”. A total number of 12 female albino Wistar rats ranging from 6 to 8 weeks were purchased from Med-Zist Company (Tehran, Iran). Animals were allowed to accommodate the condition prior to the experimental protocol. All animals were always maintained in standard cages with unlimited access to pellet food and water with the cycle of 12 h light at darkness under the standard temperature of 22 ± 2 °C.

#### Experimental design

One week post-accommodation, rats were randomly assigned into two groups (each in 6) as follows; I: control group and II: rats received 160 mg/kgbw VCD (Sigma, 94956) intraperitoneally for 15 consequent days. To administrate VCD, the drug was diluted in an appropriate solvent and injected in the final volume of 0.2 ml. Control rats were injected with 0.2 ml of VCD-free normal saline.

#### Sampling

To address the possible effect of VCD on ovarian, hepatic and renal tissues, animals were humanly euthanized by the combined regime of ketamine and xylazine. At respective time points, tissue samples were taken. Samples were rinsed with phosphate-buffered saline to eliminate excess blood and kept in 10% formalin solution (Merck) and subjected to histological analyses.

#### Histopathological examination

For this propose, following fixation, samples were embedded in paraffin solution. Subsequently, 5-µm thick sections were prepared by using a microtome (Leica). For staining, sections were incubated with H&E solution. The existence of pathologies and abnormal structures samples from liver, kidneys, and ovaries were monitored in each slide from different high power fields.

#### Biochemical examination

To examine the possible toxic effect of VCD on the systemic levels of biomarkers associated with the function of kidneys and liver, the serum contents of ALT, AST, creatinine, and urea was evaluated 2 weeks after VCD administration. To measure these markers, blood samples were let to clot and supernatant sera isolated. The levels of these biofactors were measured using biochemical kits (Zellbio, Germany) according to the manufacturer’s instruction.

#### Statistical analysis

Results of the current experiment are presented as the mean ± SD. To compare the significant levels of enzyme and biochemical factors between groups, Student t-test using GraphPad Prism was performed. p < 0.05 was considered statistically significant.

### Results

#### Histological examination

**Fig. 1:**
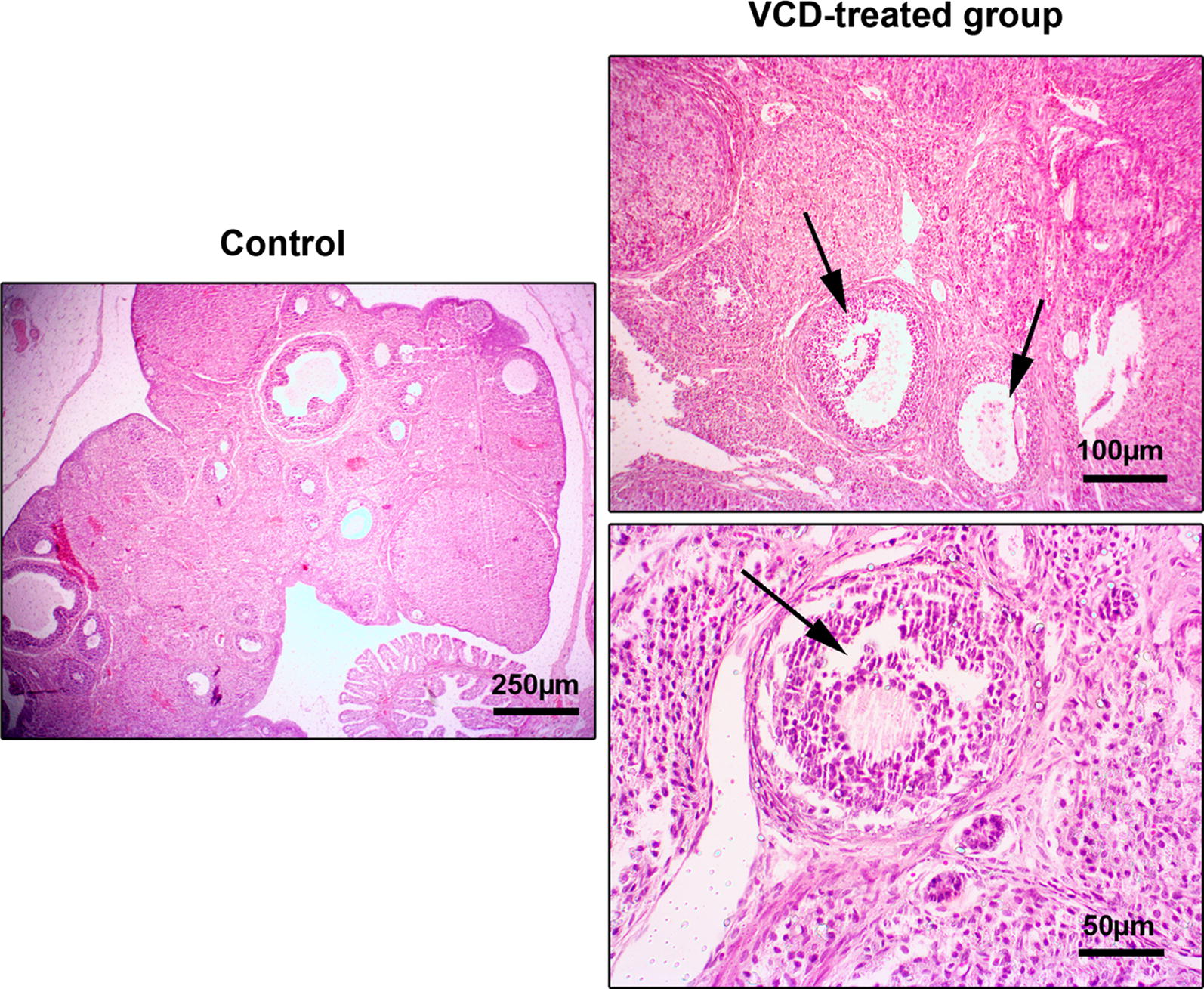
H&E staining of the ovarian tissue. In addition to hyperemia in the ovarian tissue of the VCD treated group, the presence of collagen fibers represents fibrosis of the tissue. Depending on the effect of VCD on follicles, degeneration of primary and secondary follicles is evident

**Fig. 2:**
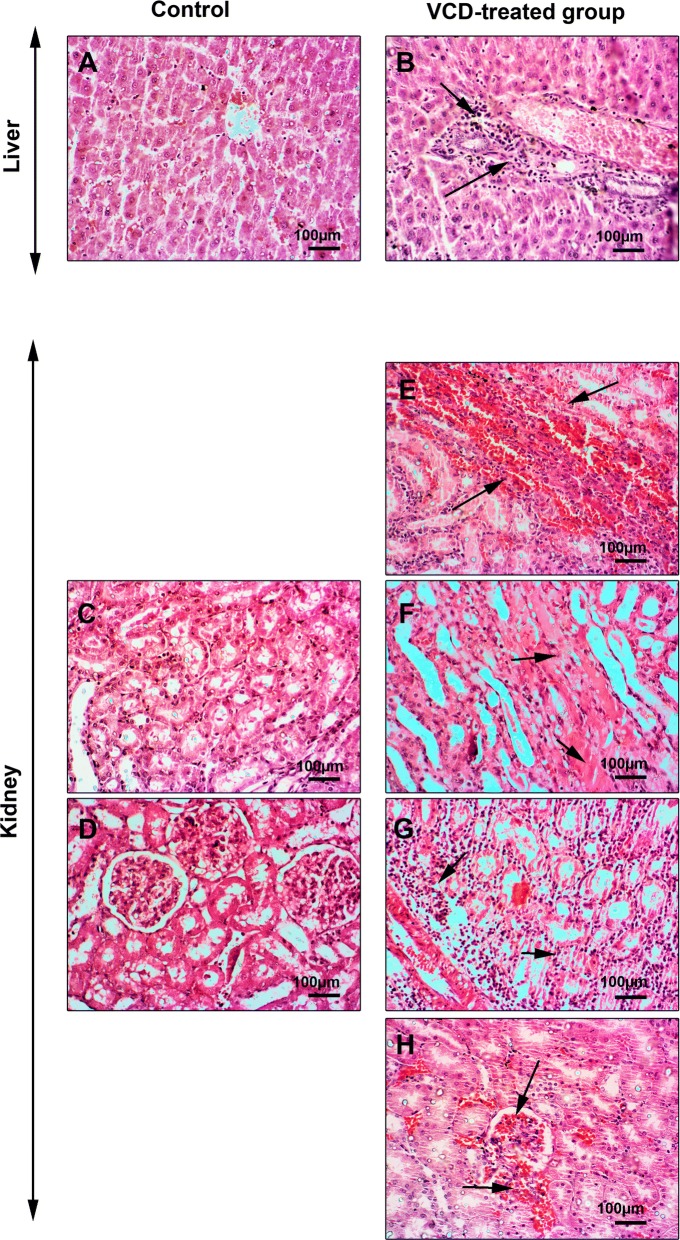
H&E staining of the rat kidney and liver tissues. Following VCD injection, hyperemia, tubular necrosis, hyaline casts, and degeneration of bowman capsules are detected. In the liver tissue, hypertension, cholangitis, and hepatocyte necrosis have been revealed to associate with VCD receiving group

Following VCD administration, a rat model of POF was produced based on ovarian tissue examination. According to data from histological analysis, we found hyperemia, and general follicular atresia, the decrease of primary and secondary follicles and detachment of oocytes from surrounding granulosa cells. These changes demonstrated the induction of menopause in rats received VCD (Fig. 1). To explore the possible toxicity of VCD in other tissues, we also monitored the histological changes of the liver and kidneys. Bright field imaging of liver and kidneys revealed the existence of tissue injuries 2-week after the use of VCD in the menopause model of rat. Data showed extensive renal tubules necrosis, focal hemorrhage, hyaline casts formation, and interstitial nephritis in VCD-treated rats (Fig. 2). In hepatic tissue, we noted numerous hemorrhagic foci, chronic cholangitis and hepatocyte necrosis, indicating intoxication in both hepatic and renal tissues (Fig. 2).

#### Serum levels of AST and ALT were increased after VCD treatment

**Fig. 3:**
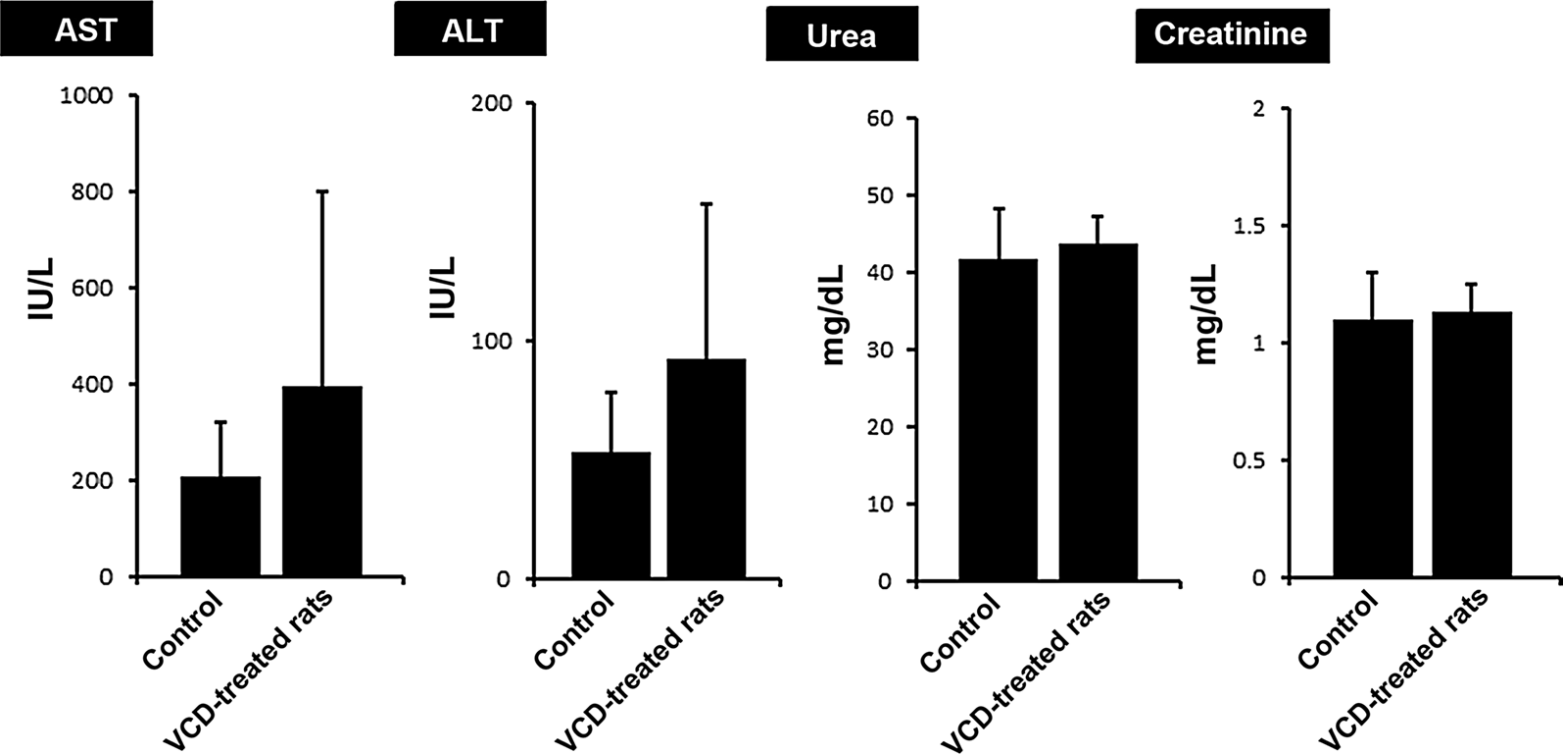
The effect of intra-peritoneal injection (IP) of VCD on kidneys and liver. Rats were treated daily for 15 days with VCD (160 mg/kgbw) and sampling from rats was done 2 weeks after final VCD injection and simultaneously blood samples processed to measure liver enzymes (AST, ALT), creatinine, and urea measured using commercial kits. In the VCD recipient group, however not significant, the hepatic enzymes had elevated in comparison with the control group also, VCD did not a significantly affected the serum levels of creatinine and urea

Based on the biochemical analysis, we found that the levels of both hepatic enzymes ALT and AST were increased in VCD group compared to control counterparts which were obtained using commercial kits (Fig. 3). However, a non-significant difference was obtained related to serum levels of hepatic tissues, ALT and AST (p > 0.05). Similar to these changes, the levels of creatinine and urea were also monitored in VCD-treated rats. Data showed a slight increase in the serum content of both AST and ALT but did not reach significant levels (p > 0.05) (Fig. 3).

### Discussion

Today, many attempts have been made to investigate the impacts of various compounds on reproduction capacity. To attain this goal, appropriate animal models have been adapted to investigate the underlying mechanisms to human counterpart. Of these compounds, VCD is a common substance used for the induction of POF status in rodent models. There is little knowledge related to the adverse effects of VCD on other organs in addition to ovaries and urogenital tract.

The current investigation was planned to elaborate the possible toxic effect of VCD on the liver and kidneys in the rat model of POF 14 days post-injection. According to our protocol, we successfully developed POF in rats as evidenced by the changes in ovarian tissue, such as follicular degeneration, atresia, granulosa cells detachment from oocytes, a decrease of follicular number, hyperemia and fibrotic changes in the ovaries in VCD-treated rats. Simultaneously, VCD exhibited toxic effects on the liver and kidneys. In this regard, a profound renal insufficiency was diagnosed that correlated with the formation of hyaline casts, hyperemia, and tubule-interstitial nephritis while in the hepatic tissue focal necrosis, hyperemia, and cholangitis were indicated. These data stand for a fact that VCD could be excreted and/or metabolized by the renal and hepatic tissues. Consistent with our data, studies conducted on female mice and rats revealed that a large portion of the administered VCD (over 50–60%) was eliminated via the renal system [16, 17]. In addition to the critical role of the renal filtration on VCD removal, liver enzymatic activity seems an alternative approach metabolizing VCD to intermediate metabolites. For instance, laboratory examination showed the conversion of VCD to different derivatives such as monoepoxymonoglycols, 1, 2-hydroxy-4-vinylcyclohexane oxide, and 4-(1′,2′-dihydroxyethyl)-1-cyclohexane oxide by liver microsomal activity in the rabbit model. Interaction with glutathione is thought of as an alternative pathway to promote the metabolism of VCD [16]. Although bioactivity of hepatic and renal systems is to eliminate VCD but high dosage administration and accumulation of VCD could cause intoxication. According to previously released data, it seems that the detrimental effects of VCD depend on the types of tissue and organ. Intra-peritoneal administration of VCD showed no adverse effects on the activity of adrenal, renal and spleen in mice and rats [18].

The function of liver and kidneys was evaluated by measuring the ALT, AST, urea and creatinine. Biochemical analysis showed that although serum levels of ALT, AST, urea and creatinine were increased compared to the control rats; however, these values did not reach statistical significant level. One reason would be that 15-day administration of VCD initiated pathological injuries but these changes are not enough to cause statistically significant differences in the haptic enzymes and the serum levels of urea and creatinine. The other reason could be that both liver and kidneys might adapt themselves to the toxicity of the VCD during a long-period treatment. Despite targeting ovarian tissue during the induction of POF by VCD in the rat model, it seems that occurrence of toxicity in vital organs such as liver and kidneys could relate to the accumulation of byproducts and toxins which indirectly affect the physiological bioactivity of the ovaries. Therefore, it could be concluded that the use of selective agents is mandatory to induce POF with minimal toxicity status in other organs. As a matter of fact, determining the time, the dosage of VCD will be essential to circumvent these limitations.

Animal modeling for human disease and insufficiencies is pivotal in a sense of research and problem-based treatments. Considering incidence of subfertility complications and the rise in the number of subjects consulting infertility centers, the need for implementing a new generation of ART is utmost importance. Proper animal modeling is a science by itself and should be performed adequately. Regarding the adverse consequences involved during animal modeling procedures, precautions should be undertaken to minimize the unwanted side-effects.

### Conclusion

VCD had adverse effects on liver and kidneys during the establishment of POF animal model. It seems that future experimentations should consider other possible chemicals/substances with less unwanted consequences.

## Limitations

Long-term studies could reflect the real state of the toxicity on other organs. In addition, future experimentations could study and optimize the effective and non-toxic concentrations of VCD during animal modeling.

## Data Availability

All data generated or analyzed during this study are included in this published article.

## Abbreviations

VCD: vinylcyclohexene dioxide
POF: premature ovarian failure
HRT: hormone replacement therapy
GnRHa: gonadotropin-releasing hormone agonist
ART: assisted reproductive technologies
VCH: 4-vinylcyclohexene
KITLG: KIT ligand
PI3 K: phosphatidylinositol 3-kinase
PDK-1: phosphoinositide-dependent protein kinase-1
ALT: alanine transaminase
AST: aspartate transaminase
H&E: hematoxylin and eosin
kgbw: kilogram body weight
